# Characterisation and Quenching Correction for an Al_2_O_3_:C Optical Fibre Real Time System in Therapeutic Proton, Helium, and Carbon-Charged Beams

**DOI:** 10.3390/s22239178

**Published:** 2022-11-25

**Authors:** Luana de Freitas Nascimento, Paul Leblans, Brent van der Heyden, Mark Akselrod, Jo Goossens, Luis Enrique Correa Rocha, Ana Vaniqui, Dirk Verellen

**Affiliations:** 1 Belgian Nuclear Research Centre, SCK CEN, 2400 Mol, Belgium; 2 Agfa NV, 2640 Mortsel, Belgium; 3 Landauer, Stillwater Crystal Growth Division, Stillwater, OK 74074, USA; 4 Faculty of Medicine and Health Sciences, University of Antwerp, 2610 Antwerp, Belgium; 5 Iridium Netwerk, University of Antwerp, 2610 Antwerp, Belgium; 6 Department of Economics, Ghent University, 9000 Ghent, Belgium; 7 Department of Physics and Astronomy, Ghent University, 9000 Ghent, Belgium

**Keywords:** real time dosimetry, hadron therapy, quenching correction

## Abstract

Real time radioluminescence fibre-based detectors were investigated for application in proton, helium, and carbon therapy dosimetry. The Al_2_O_3_:C probes are made of one single crystal (1 mm) and two droplets of micro powder in two sizes (38 μm and 4 μm) mixed with a water-equivalent binder. The fibres were irradiated behind different thicknesses of solid slabs, and the Bragg curves presented a quenching effect attributed to the nonlinear response of the radioluminescence (RL) signal as a function of linear energy transfer (LET). Experimental data and Monte Carlo simulations were utilised to acquire a quenching correction method, adapted from Birks’ formulation, to restore the linear dose–response for particle therapy beams. The method for quenching correction was applied and yielded the best results for the ‘4 μm’ optical fibre probe, with an agreement at the Bragg peak of 1.4% (160 MeV), and 1.5% (230 MeV) for proton-charged particles; 2.4% (150 MeV/u) for helium-charged particles and of 4.8% (290 MeV/u) and 2.9% (400 MeV/u) for the carbon-charged particles. The most substantial deviations for the ‘4 μm’ optical fibre probe were found at the falloff regions, with ~3% (protons), ~5% (helium) and 6% (carbon).

## 1. Introduction

Particle therapy has gained popularity as an effective technique for cancer treatment due to its greater precision in dose delivery and less damage to adjacent healthy tissue and organs. The complexity of particle therapy brings several technical challenges for dosimetrists, radiobiologists, and medical physicists. In order to evaluate the performance of particle therapy against conventional radiotherapy, it is essential to test and report the response of various treatment modalities using similar methods across treatment and research centres. International agencies (e.g., the International Atomic Energy Agency-IAEA, the American Association of Physicists in Medicine-AAPM, and the International Commission on Radiation Units and Measurements-ICRU) have been working on global standard protocols to harmonise the reporting of treatments. The standards should cover beam production, dosimetry, relative biological effectiveness (RBE), treatment planning, clinical requirements and protocols [1,2].

According to the report “Dose Reporting in Ion Beam Therapy” proposed by the IAEA (Report No. 1560) [3], to guarantee a cost-effective operation of a therapy facility, the time required for the quality assurance (QA) program must be kept to a minimum while ensuring complete coverage of all critical system parameters. Each particle therapy centre worldwide should thus optimise its QA procedure.

When looking specifically at dosimetry, several authors have published on charged particle QA using either commercial or in-house developed devices [4,5,6,7]. Most of the systems in use are based on single ionisation chambers or diodes, or arrays of them, and on radiographic/radiochromic films, which can be considered sufficiently precise but in need of lengthy procedures and, in the case of diodes and films, as having energy-dependent effects with linear energy transfer (LET) [8,9,10,11,12,13]. The quantity of LET describes the average energy transfer from electronic interactions per unit length travelled by charged primary particles.

One class of promising dosimeters is based on luminescence detectors. One advantage is the diverse (point, 1D, 2D) readout possibilities, both as active (radioluminescence-RL, scintillation) and as passive detectors (thermoluminescence-TL, optically stimulated luminescence-OSL and radiophotoluminescence-RPL) [5,14,15,16,17,18,19,20]. One known drawback of luminescence detectors is the existence of the quenching effect [21], which is the dose nonlinearity effect caused by the high ionisation density in particles of high LET, i.e., the higher the particle LET, the lower the light production efficiency from the luminescence detector, resulting in substantially under-estimated doses [22,23,24,25,26,27].

In this paper, we further explore a novel quenching correction method to restore the linear dose–response for particle therapy beams. Our approach builds on a method previously used to correct for quenching in plastic scintillator detectors using the Birks equation [28,29]. The novelty of our method is to include the contribution from fragments generated along the Bragg curves by proposing a novel general expression for quenching correction factors. In a previous attempt to correct the response to proton and carbon-charged particle beams in 2D, real time RL films, we observed that a better agreement could be reached by including more fragments in our calculations [30].

The aforementioned method [30] is now applied on three types of RL optical fibre probes, composed of Al_2_O_3_:C, in five therapeutic charged particle beams: 160 and 230 MeV protons, 150 MeV/u helium ions, and 290 and 400 MeV/u carbon ions. For all the optical fibre probe types, the quenching-corrected doses along the Bragg curves resulted in substantial improvement when compared to uncorrected data.

## 2. Materials and Methods

### 2.1. Irradiations at HIMAC and SCK CEN

**Figure 1:**
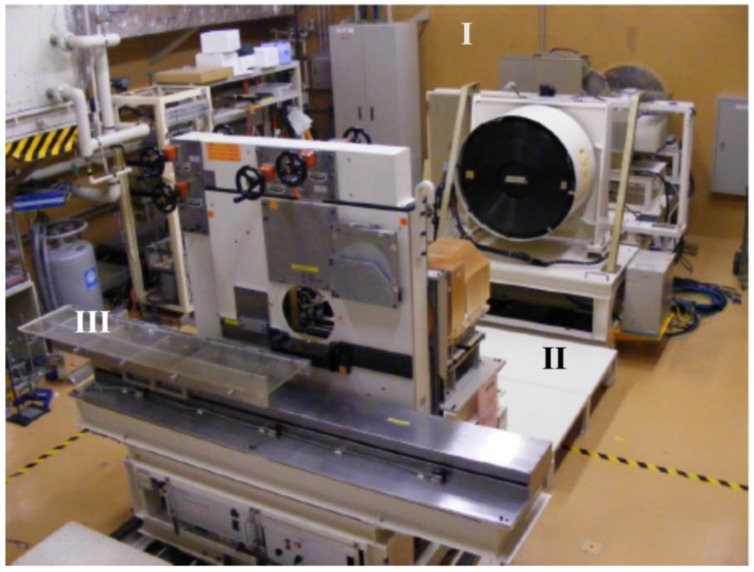
Biological experiment room (BIO). The area around “I” indicates the horizontal beam line, “II” indicates the set of binary filters, and “III” is the position of the fibre probes.

The Heavy Ion Medical Accelerator facility (HIMAC) in Chiba, Japan, consists of three clinical treatment rooms, one biological experiment room (BIO), two large general experimental halls, and a low-energy experimental room. Figure 1 shows the BIO room with a horizontal beam line (indicated by (I)), a pair of wobbler magnets and a scatterer used to produce uniform irradiation fields. The range shifter is used for adjusting the residual range of the heavy ions in the target. A set of binary filters changes the depth in the measurements (II).

**Figure 2:**
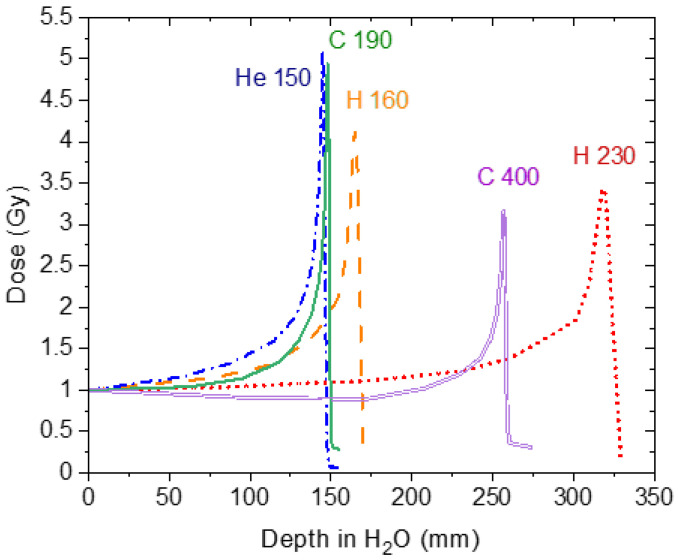
Bragg curves from 160 and 230 MeV proton, 150 MeV/u helium and 290 and 400 MeV/n carbon mono-energetic beams measured with reference ion chambers.

For our experiments, we irradiated optical fibres coupled to Al_2_O_3_:C sensors with 160 and 230 MeV proton, 150 MeV/u helium, and 290 and 400 MeV/u carbon mono-energetic beams. Actual energies, based on reference measured Bragg curves and Monte Carlo simulations, were 154.5 MeV (LETw = 5.33 MeV/cm) and 226.5 MeV (LETw = 4.15 MeV/cm) protons, 142.6 MeV/u (LETw = 22.44 MeV/cm) helium ions, and 273.8 MeV/u (LETw = 132.9 MeV/cm) and 383.2 MeV/u (LETw = 111.1 MeV/cm) carbon ions (Figure 2). The experimental set-up consisted in positioning Al_2_O_3_:C+fibre probes in front of a Polymethylmethacrylate (PMMA, density = 1.19 g cm^−3^) binary filter (“III” in Figure 1) with different water-equivalent thicknesses (depth in H_2_O.). The filters consist of 9 plates of PMMA of 0.5, 1, 2, 4, 8, 16, 32, 64, and 128 mm thickness, covering a circular 10 cm radiation field.

Reference data (absorbed doses and depth-dose profiles) were acquired using a Markus ionisation chamber [31,32] (Figure 2). The given absorbed doses to water and dose rates for each beam/energy type are summarised in Table 1. Bragg curves had different depths in water within a 10 cm × 10 cm lateral field, with flatness better than 3%.

Supplementary dose reference data were obtained using ^60^Co gamma-ray irradiator Theratron 780 at the Belgian Laboratory for Standard Dosimetry Calibrations (LNK, SCK CEN, Belgium) [33]. The fibre probes were exposed with a reference nominal dose rate of 0.5 Gy/min and a total dose of 1.0 Gy ($D_{Co}$).

These reference irradiations with ^60^Co gamma beams were used to compare the RL signal with high LET ($RL_{LET}$) to the signals with low LET from photons ($RL_{Co}$) and to calculate the luminescence efficiency further. Reference irradiations took place before and after the measurements at HIMAC to account for any changes in material sensitivity.

The relative luminescence efficiency *μ* used in this paper was previously employed by Sawakuchi et al. [34] and Kalef-Ezra and Horowitz [35]. It is defined as the ratio of the luminescence signal to the radiation field *k*, and the luminescence signal to a reference radiation field *l*, for a specific dose D_k_ or D_l_. Equation (1) gives the relative luminescence efficiency *μ* for heavy charged particles (HCP) and ^60^Co gamma rays.

(1) $$\mu =\frac{RL_{HCP}/D_{HCP}}{RL_{Co}/D_{Co}}=C\cdot RL_{HCP}/D_{HCP}$$

where *RL_HCP_* and *RL_Co_* are the measured luminescence signals (RL), and *D_HCP_* and *D_Co_* are the absorbed doses, respectively, from the irradiations with heavy charged particles (HCP) and ^60^Co gamma rays. The quantity ${\mathrm{RL}}_{\mathrm{Co}}/D_{\mathrm{Co}}$ is a constant (*C*) for each fibre type.

### 2.2. Optical Fibres and Detectors

We tested several fibre probes with Al_2_O_3_:C crystals and powder grains produced by Landauer, Stillwater, OK, USA: one ‘Single Crystal’-type, with one Al_2_O_3_:C crystal (2 × 1 × 1 mm^3^); two ‘38 μm’-types, with droplets containing Al_2_O_3_:C with average micro-crystal (or grain) size of 38 μm [36,37] (*r* = 0.5 mm and *l* = 200 μm); and two ‘4 μm’-types, with droplets containing Al_2_O_3_:C with an average crystalline grain size of 4 μm (*r* = 0.5 mm and *l* = 200 μm) [38]. All PMMA optical fibres were 15 m long, with a 1 mm diameter. We irradiated the detectors before the experiments in HIMAC to fill deep traps to saturation [39]. A bi-alkali photomultiplier tube (PMT) P30USB (Sens-Tech^TM^) reads the RL signal from the probes, while two 2 mm 425 nm Hard Coated Broadband Bandpass Interference Filters (Edmund Optics, Nether Poppleton, York, United Kingdom) allow only the slow 420 nm component from Al_2_O_3_:C to pass. For all measurements, we sampled at 200 points per second using a NI USB 6341 DAQ card (National Instruments, Austin, TX 78759, USA) for data acquisition and control via an in-house developed LabVIEW software. More details of the RL prototype can be found in previous publications [38,40,41].

The evaluation of the relative efficiency μ by Equation (1) requires the doses D_HCP_ and *D_Co_* to be in the linear range of the dose–response. In order to check if the *RL_HCP_* is linearly proportional to *D_HCP_*, the fibres were irradiated with nominal doses *D_HCP_* (in water) in the ranges presented in Table 1 for each beam type, energy, dose rate, and type and quantity of fibre probes.

### 2.3. Quenching Correction

To correct the fibre’s measured luminescence for quenching along the central axis of the particle beams, we used the Birks law, further adapting the method proposed by Robertson et al. and Almurayshid et al. [29,42,43] by combining the contribution of fragments along the Bragg curve and the relative luminescence efficiency (μ) described in Section 2.1.

The Birks model describes the RL light emission in terms of the stopping power of the phosphor for the particle beam, according to Equation (2) below.

(2) $$\frac{\mathrm{dRL}}{\mathrm{dx}}={\mathrm{RL}}_{0}\cdot \frac{\mathrm{dE}/\mathrm{dx}}{1+\mathrm{kB}\cdot \mathrm{dE}/\mathrm{dx}}$$

where RL is the luminescence intensity, *dE*/*dx* is the specific energy deposited by the particles per unit of path length *x* in the medium, kB is the Birks constant (μg MeV^−1^ cm^−2^), which depends on the charged particle type and the material and *RL*_0_ is the relative luminescence efficiency of the medium. We rewrite Equation (2) in terms of finite voxels to describe a more realistic therapeutic charged particle beam, where we replace the stopping power term of the Birks equation with LET [44] as follows (Equation (3)):

(3) $${\mathrm{RL}}_{v}=\left(\frac{{\mathrm{RL}}_{0}\cdot {\mathrm{LET}}_{v}}{1+\mathrm{kB}\cdot {\mathrm{LET}}_{v}}\right)\cdot \phi_{v}$$

where *RL_v_* is the light emitted from a voxel of volume “v” (Al_2_O_3_:C droplets or crystal described in Section 2.2), LET_v_ is the fluence-averaged LET within the voxel, and $\phi_{v}$ is the particle fluence in the voxel. The fluence and fluence averaged-LET (LET_f_) from the nuclear fragments of the primary beams were generated via the “TOol for PArticle Simulation” (TOPAS) Monte Carlo code [45] Monte Carlo calculations (Section 2.4).

The finite size of the active volume in the Al_2_O_3_:C probes caused an averaging of the dose gradients along the Bragg curves. The dose and LET are scored in 0.1 mm volumes in TOPAS, while the probes have different volumes, as described in Section 2.2. The deviation between the dose and LET scored in such voxels compared to the same quantities scored in 0.1 mm wide voxels is taken into account and corrected.

In Equation (3), RL_v_ = RL_HCP_, gives a direct link between the measurements with the fibre probes (‘RL signal’), the nominal given doses measured with the reference Markus chamber (D_HCP_), and the Birks law for quenching.

In order to correct the measured dose for quenching, a correction factor η is required. This factor takes the form of Equation (4), where the ratio of deposited energy (*E_v_*) to the emitted *RL_v_* light in the voxel “v” can be expressed as:

(4) $$\eta_{v}=\left(\frac{E_{v}}{RL_{v}}\right)=\left(\frac{\phi_{v}\cdot LET_{v}}{RL_{v}}\right)=\frac{1+kB\cdot LET_{v}}{RL_{o}}$$

Our proposed quenching correction factor η [30] is the sum of the fluence-weighted quenching corrections η_i_ for each particle type (primary and fragment) for specific LET ranges in water.

(5) $$\eta =\sum_{d=0}^{n}\sum_{i}\left(f_{d}^{i}\cdot \eta_{i}\right)=\sum_{d=0}^{n}\sum_{i}\left[f_{d}^{i}\cdot \left(\frac{1+kB_{i}\cdot LET_{d}^{i}}{RL_{o_{i}}}\right)\right]$$

where $f_{d}^{i}$ is the percentage contribution in fluence of particle “*i*” at position “*d*”, multiplied by the correction factor corresponding to the LET at depth *d*. Each particle (primary and fragment) has its own Birks constant kB_i_ and multiplication factor 1/$RL_{o_{i}}$.

We determined the Birks constant, kB and the relative luminescence efficiency RL_0_ for each fibre type (single crystal, 38 and 4 µm powder) by plotting the normalised ‘RL signal’ = [(μ$\cdot D_{{HCP}_{i}}$)/${DHCP}_{entrance}]$ (*i* = depth in H_2_O) versus LET_f_ (TOPAS) and then fitting the curves by using Equation (3) in the nonlinear curve fit option in the “fitting” routine in Origin(Pro) (Version 2020b, OriginLab Corporation, Northampton, MA, USA). The parameter RL_o_ is a scaling factor dependent on the detector geometry and the fluence in the Monte Carlo calculation, while the Birks constant unit is mg·MeV^−1^cm^−2^.

By applying the quenching correction factors to all ‘RL signal’ along the Bragg curves, weighted by the relative luminescence efficiency (that correlates ‘RL signal’ to reference D_HCP_), one obtains a corrected dose distribution for each fibre probe type.

### 2.4. Monte Carlo Simulations

The LET values used to correct the quenching from the optical fibre probes are based on fluence-averaged LET (LET_f_). Fluence-based (LET_f_) and dose-based (LET_D_) LET values can vary considerably with depth [46] according to the choice of step limit. This effect strongly affects the LET_D_ for small step sizes (<500 µm) because Monte Carlo codes usually only consider collisions where the kinetic energy imparted to secondary electrons is below a given threshold, restricting the quantity to shorter-range electrons and giving better characterisation when one wants to correlate the radiation effects to RBE or microdosimetry [47]. This step-limiting effect was studied by Guan et al. [46,48] and further addressed and used by other authors [18,29,49,50,51]. The agreement is that the step limit effect is negligible for LET_f_ although it strongly affects LET_D_ results [52,53]. Since the size of the detectors used in our study is not at the cellular scale (μm), we decided to show only the results related to LET_f_.

The “TOol for PArticle Simulation” (TOPAS) Monte Carlo code [45] was used to simulate the fluence and LET_f_ from the primary beams and their nuclear fragments. The proton, helium, and carbon ion simulations were performed respectively with 10*10^6^, 20*10^6^, and 25*10^5^ histories. The error statistics in output results (fluence) were (a) <0.01% along the 160 MeV proton beam up to the Bragg peak (0.04% at the 80% distal falloff depth), (b) <0.02% along the 230 MeV proton beam up to the Bragg peak (0.04% at the 80% distal falloff depth), (c) <0.01% along the 150 MeV/u helium beam up to the Bragg peak (0.07% at the 80% distal falloff depth), (d) <0.05% along the 290 MeV/u carbon beam up to the Bragg peak (0.55% at the 80% distal falloff depth), and (e) <0.1% along the 400 MeV/u carbon beam up to the Bragg peak (0.2% at the 80% distal falloff depth). In TOPAS, a particle fluence scorer and a fluence-averaged LET scorer were attached to the simulated water volume in function of beam penetration depth. A dedicated filter was assigned to both active scorers to separate the scored fluence and LET signals for the primary beam (^1^H, ^4^He or ^12^C) and a list of nuclear fragments (^1^H, ^4^He, ^6^Li, ^7^Be, ^10^B, ^14^N and ^16^O). In post-processing software, written in Matlab R2020b (The Mathworks Inc., Natick, MA, USA), the output of the energy deposit scorer was divided by the output of the fluence scorer, multiplied by the voxel volume to obtain LET in MeV/mm units. The final LET values were converted to MeV/cm or keV/μm. Fluence and fluence LET were scored with the resolution of 0.1 mm, so that the entrance position for TOPAS simulations is defined as within the first 0.1 mm in water.

### 2.5. Overview of Tests

The dose–response curves of proton, helium, and carbon-charged particles were evaluated by placing the fibre probes at entrance depth, where each dose relates to the ‘RL signal’ in the beam’s isocenter, as defined in Section 2.1 and Figure 1. The RL emission from Al_2_O_3_:C is proportional to the dose rate [38,40]. To correlate ‘RL signal’ to the given dose, the ‘ΔRL’ is calculated as the sum of the ‘RL signal’ from the start of irradiation (*t* =  0) until the end (*t* = T), corrected for the averaged background ($\bar{Bkg}$) for each independent irradiation (Equation (6)). Each $\bar{Bkg}$ was acquired by averaging 25 measured points prior and 25 measured points post-irradiation (*m* = 50).

(6) $${\prime \mathrm{RL}\;\mathrm{signal}}^{\prime }=\Delta RL=\sum_{t=0}^{t=T}RL_{t}-\left[\frac{1}{m}\cdot \sum_{n=1}^{m}Bkg_{n}\right]=\sum_{t=0}^{t=T}RL_{t}-\bar{Bkg}$$

The linearity of the dose response was evaluated by calculating the linearity index of the measurements. The linearity index describes the departure of the detector’s response from linearity at a chosen calibration dose D_0_. The sensitivity of the fibre probe at dose D_i_ for the i-th radiation is related to the observed RL signal (‘RL signal’_i_/D_i_) and is further normalised by the chosen calibration dose D_0_ = 1 Gy so that the linearity index is: (‘RL signal’_i_/D_i_)/(‘RL signal’_1 Gy_/D_1 Gy_). Fitting curves were derived to correct further the ‘RL signal’ for the dose ranges where deviations from linearity were observed (Section 3.1).

The Al_2_O_3_:C optical fibre probes’ dependence on radiation LET was assessed by measuring the Bragg curves for proton, helium, and carbon-charged particles. The measurements in the beam’s isocenter were rescaled according to the relative luminescence efficiencies at entrance doses (d in H_2_O = 0 mm) and compared with the curves assessed with a Markus ionisation chamber in terms of peak-to-plateau ratio signals (Section 3.2). The Monte Carlo simulated fluence and fluence-averaged LET (Section 3.3) was used to correct the depth-dose curves for quenching using the quenching model for proton, helium and carbon-charged particles determined in Section 2.3. The model was first implemented assuming only the primary proton-charged particles and later expanded considering a combination of the primary beam plus fragments for the helium (^1^H fragment) and carbon- (^1^H and ^4^He fragments) charged particles (Section 3.3 and Section 3.5).

## 3. Results

In this Section, we present the results and analysis of the measurement campaigns, starting with the dose responses and Bragg curves for all probe types, followed by the determination of calibration curves for the ${\;\mathrm{and}\;\eta }_{\mathrm{LET},\mathrm{Co}}$ compared to LET. We introduce a method to determine the unknown doses and LET of particle therapy fields using a combination of two or more probes. We used the LET dependence of the RL from different Al_2_O_3_:C probes to establish fluence-LET (LET_f_) calibration curves. Our fundamental assumption was that the RL signal does not depend on beam type/energy, as well as dose–rate and absorbed dose, and thus the RL signal can describe averaged LET values. Our results offer a proof of concept of the proposed method. Limitations on applying this method in practical applications will be discussed at the end of this session.

### 3.1. Fibres Dose Response

**Figure 3:**
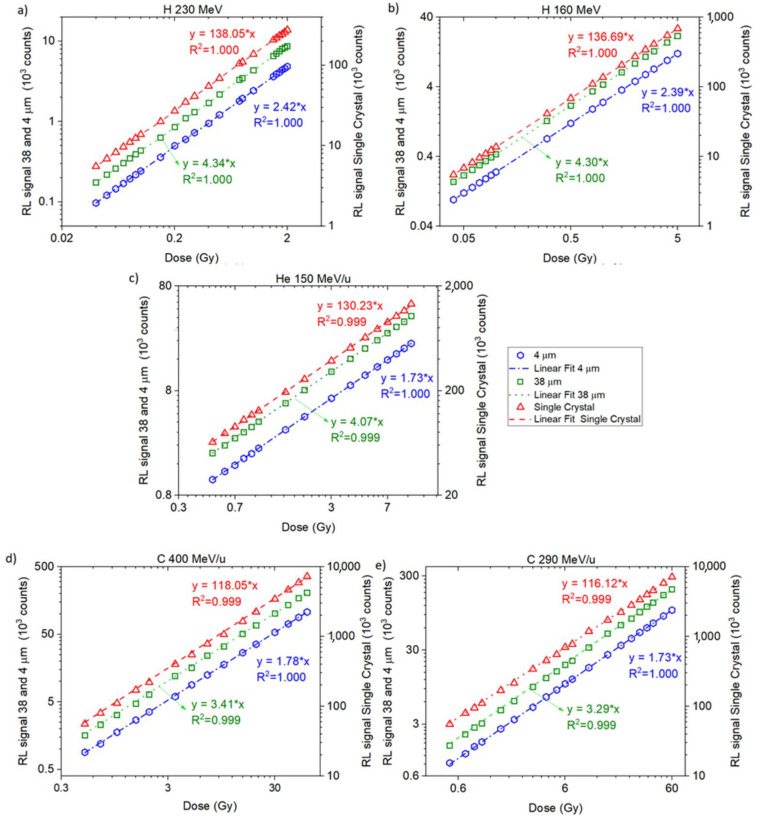
Dose response ‘Single Crystal’, ‘38 μm’ and ‘4 μm’ from (**a**) 0.04 to 2.00 Gy (0.08 Gy/min) and from 0.1 to 0.5 Gy (0.25 Gy/min) irradiated with 160 MeV protons; (**b**) 0.03 to 0.5 Gy (0.36 Gy/min) and from 0.5 to 4.0 Gy (0.65 Gy/min) irradiated with 230 MeV protons; (**c**) 0.3 to 10.0 Gy (4.5 Gy/min) irradiated with 150 MeV/u helium ions; (**d**) 0.5 to 10.0 Gy (3.04 and 7.34 Gy/min) irradiated with 290 MeV/u carbon ions; (**e**) 0.1 to 5.0 Gy (6.90 Gy/min) irradiated with 400 MeV/u carbon ions.

Figure 3a–e show the dose calculated from the ΔRL for ‘Single Crystal’, ‘38 μm’ and ‘4 μm’ fibre probes irradiated with 160 MeV proton, 230 MeV proton, 150 MeV/u helium, 290 MeV/u carbon and 400 MeV/u carbon, respectively. In all figures, each point is the average of independent irradiations (Table 1), and the standard deviations (1 SD), not plotted in the graphs, are below 1% for 38 and 4 μm fibres and 5% for the ‘Single Crystal’ probe.

The proton curves are very similar for all the same fibre types, with slopes (s) of 2.42 and 2.39 (‘4 μm’), 4.34 and 4.30 (‘38 μm’), and 138.05 and 136.69 (‘Single Crystal’) for 230 and 160 MeV, respectively, resulting in $\bar{s}$ = 2.405 ± 0.015 (4 μm), $\bar{s}$ = 4.32 ± 0.02 (38 μm), and $\bar{s}$ = 137.37 ± 0.68 (‘Single Crystal’). The higher the beam LET, the flatter the curves.

Before measuring the RL signal along the Bragg curve, we tested in which dose ranges the optical probes responded linearly. The available dose rates for irradiations with both 160 and 230 MeV protons were considerably lower than those available for helium and carbon (Table 1). Hence, the dose ranges in Figure 3 are different.

**Figure 4:**
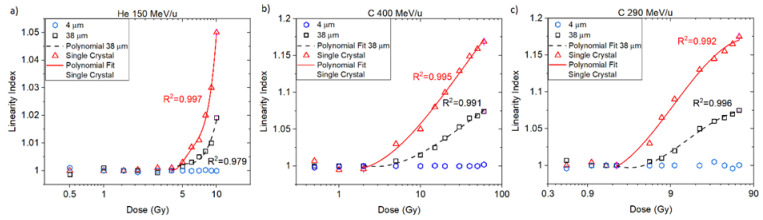
Linearity index relative to 1 Gy for ‘4 μm’, ‘38 µm’ and ‘single crystal’ optical fibre probes for (**a**) He 150 MeV/u, (**b**) C 400 MeV/u, and (**c**) C 290 MeV/u charged particles.

The linearity index is depicted in Figure 4 for the helium and carbon beams. A supralinear behaviour is observed for both ‘38 μm’ and ‘Single Crystal’ starting from 2 Gy, with maximum overresponses of 7.5% (‘38 μm’) and 17.5% (‘Single Crystal’) at 60 Gy for the carbon-heavy charged particles. The linearity index for the ‘4 μm’ fibre probe did not show supralinearity for doses below 60 Gy, and no correction was needed for the subsequent results.

The nonlinear response in the ‘38 μm’ and ‘Single Crystal’ curves were corrected by fitting the linearity index as a function of dose (D). A linearity correction factor (LCF) was defined for each ‘38 μm’ and ‘Single Crystal’ curve as shown in Equation (7) below and was applied in subsequent sections to correct for the doses measured along the Bragg curves.


(7) $$\left\{\begin{array}{c} {LCF}_{SingleCrystal}^{He\;150}\;=\;8.11\;\cdot \;D^{4}\;-\;24.38\;\cdot \;D^{3}\;+\;27.46\;\cdot \;D^{2}\;-\;13.66\;\cdot \;D+3.53\\ {LCF}_{38\mu m}^{He\;150}\;=\;0.82\;\cdot \;D^{3}\;-\;1.83\;\cdot \;D^{2}\;-\;1.36\;\cdot \;D+0.66\\ {LCF}_{SingleCrystal}^{C\;400}\;=\;-0.03\;\cdot \;D^{3}\;+\;0.12\;\cdot \;D^{2}\;-\;0.03\;\cdot \;D+0.99\\ {LCF}_{38\mu m}^{C\;400}\;=\;-0.02\;\cdot \;D^{3}\;+\;0.09\;\cdot \;D^{2}\;-\;0.07\;\cdot \;D+1.01\\ {LCF}_{SingleCrystal}^{C\;290}\;=\;-0.06\;\cdot \;D^{3}\;+\;0.18\;\cdot \;D^{2}\;-\;0.02\;\cdot \;D+0.99\\ {LCF}_{38\mu m}^{C\;290}\;=\;-0.05\;\cdot \;D^{3}\;+\;0.19\;\cdot \;D^{2}\;-\;0.17\;\cdot \;D+1.03 \end{array}\right\}$$

### 3.2. Bragg Curves

**Figure 5:**
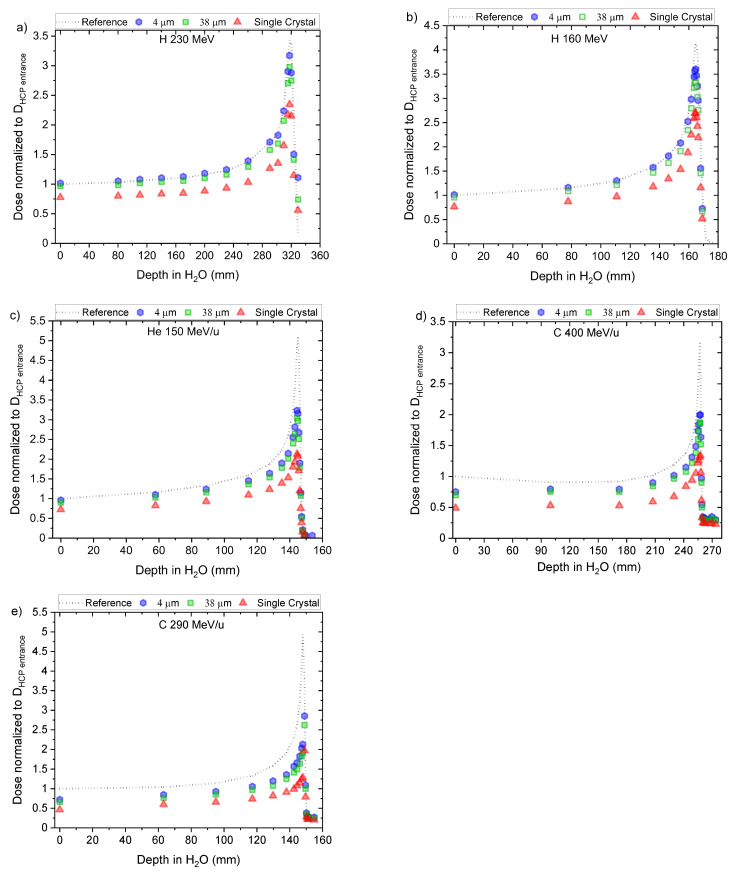
Central axis peak-to-plateau ratios (dose normalised to D_HCP_ entrance) profiles along the Bragg curves for ‘Single Crystal’, ‘38 μm’ and ‘4 μm’ probes for (**a**) H 160 MeV; (**b**) H 230 MeV; (**c**) He 150 MeV/u; (**d**) for C 400 MeV/u, and (**e**) C 290 MeV/u.

To assess the Bragg curves, we chose specific entrance doses in the linear range for each fibre type and energy (Figure 5 and Table 1) or, when necessary, used corrections according to each fibre type-dose response curve (Equation (7)).

The relative luminescence efficiencies (μ) of ‘Single Crystal’, ‘38 μm’ and ‘4 μm’ were calculated for the RL signals measured at the entrance doses (d = 0.0 mm in H_2_O) using Equation (1). Table 2 shows the calculated μ for each probe type and beam energy, corresponding to the average of different dose rate measurements (Table 1), and the error corresponds to one standard deviation (1 SD).

Using the calculated μ at entrance depth from Table 2, we rescaled the doses measured with the optical fibre probes along the Bragg curve for each beam type/energy and fibre type. Figure 5a–e shows the rescaled Bragg curves (quenched) and the ion chamber reference for the 160 and 230 MeV protons, 150 MeV/u helium ions, and 290 and 400 MeV/u carbon ions, respectively.

A comparative analysis of the calculated μ for the three types of optical probes exposed to protons, helium and carbon-charged particles indicates a larger statistical error for ‘Single Crystal’. This result is partially due to the spatial non-uniformity of an ion beam at the crystal target area (1 mm) compared to the droplet probes (0.2 mm).

### 3.3. Fluence and Fluence Averaged-Let

**Figure 6:**
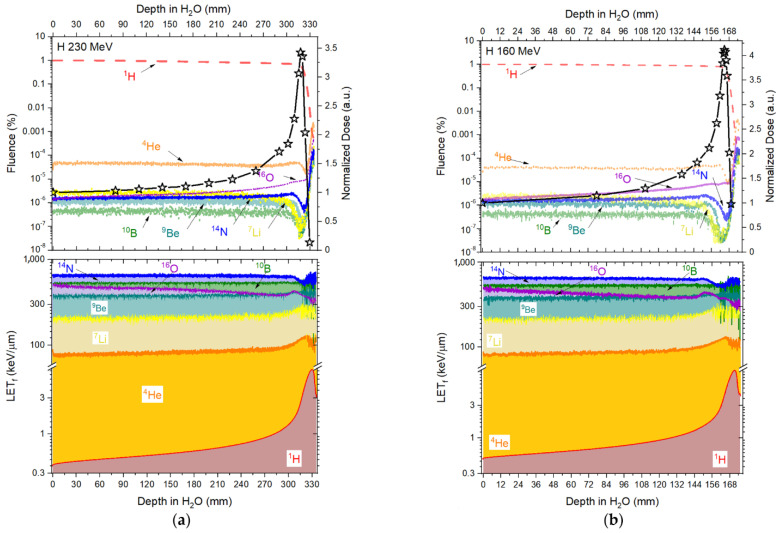
Fluence (upper-left *Y*-axis) and LET_f_ (bottom-left *Y*-axis) contribution in depth in water from the primary 230 (**a**) and 160 (**b**) MeV proton-charged particles and their fragments (H, He, B, Li, Be, N and O) simulated in TOPAS. Upper-right *Y*-axis presents the reference measured doses (Markus ion chamber) normalised to the entrance dose (D_HCP_/D_HCP entrance_).

**Figure 7:**
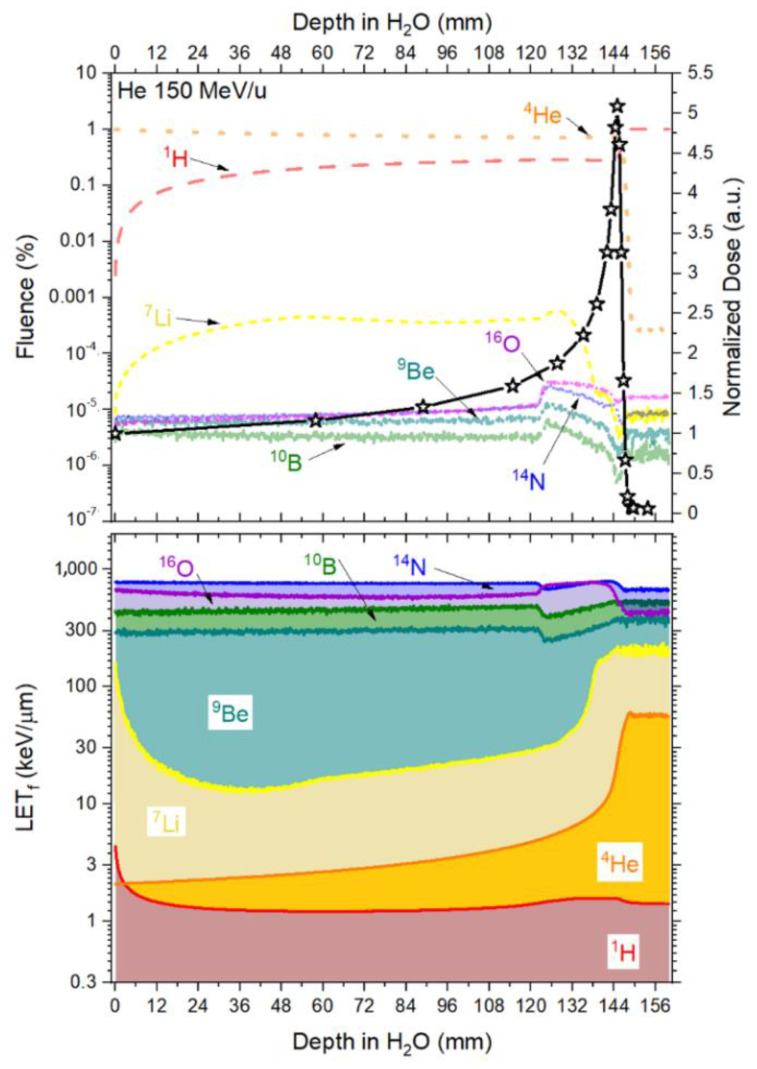
Fluence (upper-left *Y*-axis) and LET_f_ (bottom-left *Y*-axis) contribution in depth in water from the primary 150 MeV/u helium-charged particles and its fragments (H, He, B, Li, Be, N and O) simulated in TOPAS. Upper-right *Y*-axis presents the reference measured doses (Markus ion chamber) normalised to the entrance dose (D_HCP_/D_HCP entrance_).

**Figure 8:**
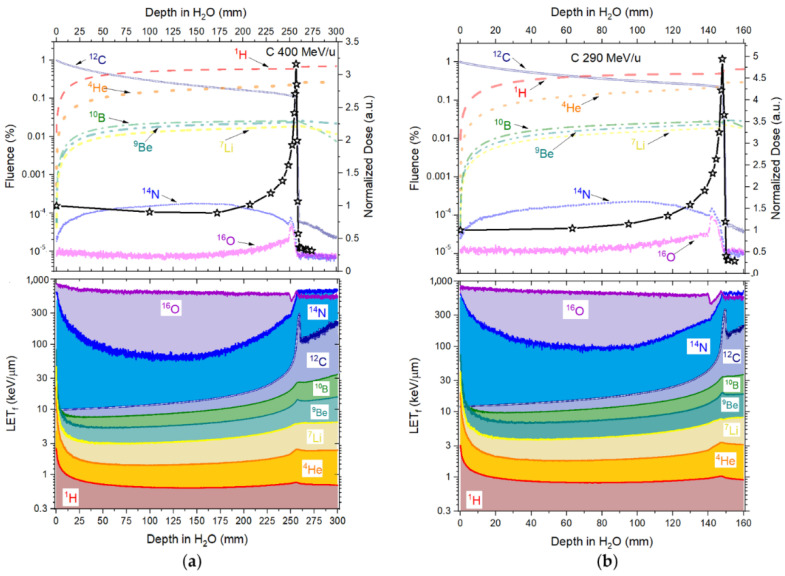
Fluence (upper-left *Y*-axis) and LET_f_ (bottom-left *Y*-axis) contribution in depth in water from the primary 400 (**a**) and 290 (**b**) MeV/u carbon-charged particles and their fragments (H, He, B, Li, Be, N and O) simulated in TOPAS. Upper-right *Y*-axis presents the reference measured doses (Markus ion chamber) normalised to the entrance dose (D_HCP_/D_HCP entrance_).

The contribution from the primary beam and its fragments along the Bragg curve concerning fluence and LET_f_, is presented in Figure 6 (protons), Figure 7 (helium ions) and Figure 8 (carbon ions).

Figure 6 and Table 3 show that the contribution, in fluence, from the primary beam (^1^H) consists of >99% along the Bragg curves for both 230 and 160 MeV. As such, we assumed that the quenching correction factors (Equation (5)) take the form of Equation (8) below and that, to correct the doses along the Bragg curves, only the primary beam Birks factors were necessary.

(8) $$\eta =\eta_{H}\left(f_{d}^{H},\;LET_{d}^{H}\right)=\left(\frac{1+kB_{1H}\cdot LET_{d}^{1H}}{R{Lo}_{1H}}\right)$$

Figure 7 and Table 4 show that the fluence coming from the primary beam decreases by around 29% at the Bragg peak (d = 30.68 mm), with 29.4% of the fluence coming from ^1^H and 0.004% coming from the other fragments. When looking at the falloff region, the contribution to the total fluence from ^1^H amounts to >98%. As such, the quenching correction factor for the Helium curves takes the form of Equation (9), where η is mostly affected by the primary beam (^4^He) and the ^1^H fragment.

(9) $$\eta =\eta_{He}\left(f_{d}^{He},LET_{d}^{He}\right)+\eta_{H}\left(f_{d}^{H},\;LET_{d}^{H}\right)=\left[f_{d}^{H}\cdot \left(\frac{1+kB_{H}\cdot LET_{d}^{H}}{RL_{o_{H}}}\right)+f_{d}^{He}\cdot \left(\frac{1+kB_{He}\cdot LET_{d}^{He}}{RL_{o_{He}}}\right)\right]$$

Figure 8 and Table 5, the primary carbon-charged particles are responsible for ~98% in fluence contribution (d = 0.01 mm), with a rapid increase in fragments contribution with deeper depths, reaching a contribution in fluence of ~61% (^1^H) and ~24% (^4^He) at the Bragg peak (d = 256.9 mm) for 400 MeV/u and for ~51% (^1^H) and ~23% (^4^He) at the Bragg peak (d = 147.92 mm) for 290 MeV/u. At the falloff region, primary carbon-charged particles are almost negligible. The quenching correction factor for the carbon-charged particles takes the form of Equation (10), where the quenching correction factor has the contribution from the primary beam (^12^C) and the ^1^H and ^4^He fragments.

(10) $$\begin{array}{cc} \eta =\eta_{C}\left(f_{d}^{C},\;LET_{d}^{C}\right) & +\eta_{He}\left(f_{d}^{He},LET_{d}^{He}\right)+\eta_{H}\left(f_{d}^{H},\;LET_{d}^{H}\right)\\ & =\left[f_{d}^{H}\cdot \left(\frac{1+kB_{H}\cdot LET_{d}^{H}}{RL_{o_{H}}}\right)+f_{d}^{He}\cdot \left(\frac{1+kB_{He}\cdot LET_{d}^{He}}{RL_{o_{He}}}\right)+f_{d}^{C}\cdot \left(\frac{1+kB_{C}\cdot LET_{d}^{C}}{RL_{o_{C}}}\right)\right] \end{array}$$

### 3.4. Relative Luminescence Efficiency Curves (μ)

**Figure 9:**
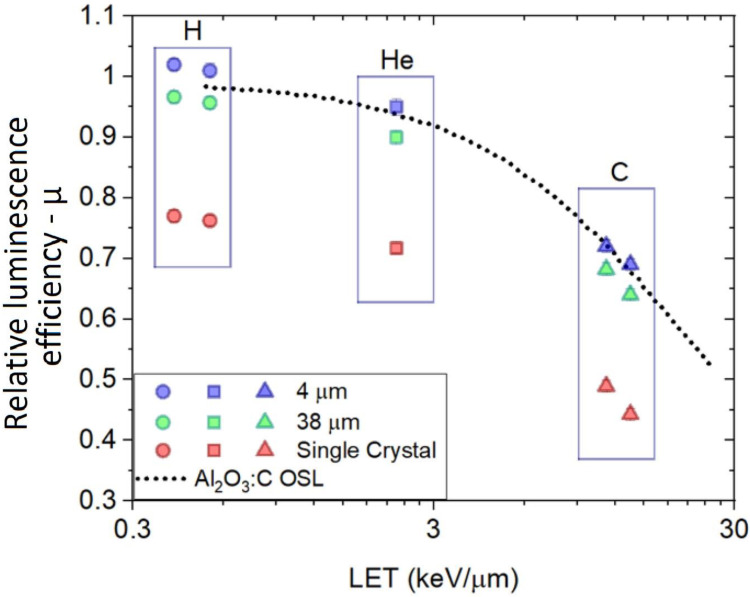
Relative luminescence efficiencies (µ) of fibre probes (‘Single Crystal’, “38 μm” and “4 μm”) at depth in H_2_O = 0 mm compared to Al_2_O_3_:C OSL results.

Figure 9 shows the calculated relative luminescence efficiency (μ) of all the optical probes at entrance depth (d = 0.01 mm) compared with data provided by Yukihara et al. using Al_2_O_3_:C OSL crystals mixed with a binder to form detectors with a diameter of 7 mm and thickness of 0.3 mm [54]. These detectors were read out, such as the LET dependence was acquired by combining the two known OSL emissions (called UV and blue). One can observe that the Al_2_O_3_:C RL and OSL relative luminescence efficiencies do follow the same decay trend, with the ‘4 μm’ showing the closest agreement with the Al_2_O_3_:C OSL.

**Figure 10:**
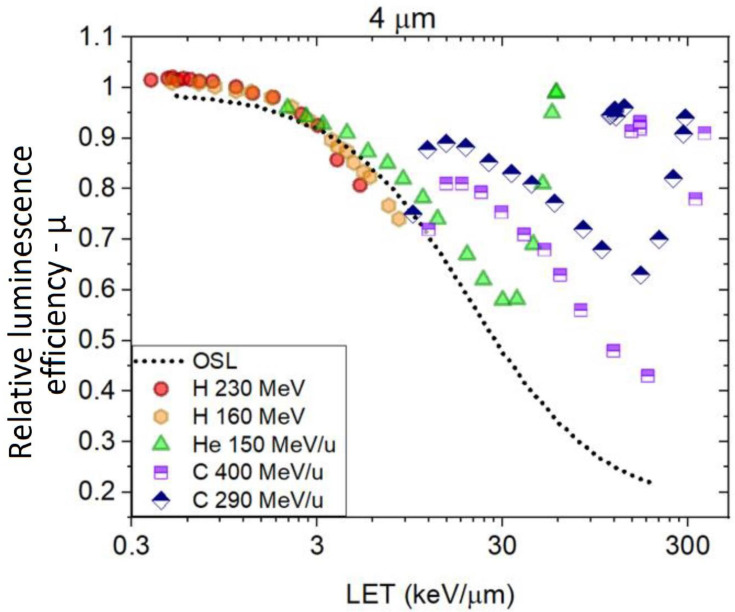
Combination of all the ‘4 μm’ Relative luminescence efficiencies (µ) acquired from different beam types and energies vs. primary LET_f_.

As a next step, we calculated the relative luminescence efficiencies along the Bragg curves and plotted the results against the simulated primary LET_f_ at each depth position. In Figure 10, the μ curve from the ‘4 μm’ fibre clearly follows the same trend as observed for the Al_2_O_3_:C OSL for the μ calculated using the primary LET_f_ from 160 (orange hexagons) and 230 (red circles) MeV proton-charged particles. At deeper depths, the μ calculated using the primary LET_f_ from 150 MeV/u helium-charged particles (green triangles) decreased to 30 keV/μm, where the curve started increasing again. For the data calculated using the primary LET_f_ from the 400 (purple squares) and 290 (blue rhombi) MeV/u carbon-charged particles, one observes first a jump from μ calculated at the entrance, with the following points following a similar trend as observed for the primary helium-charged particles.

Similar results were observed for the ´38 μm´ and ´Single Crystal´ fibre probes when plotting the relative luminescence efficiencies along the Bragg curves vs. simulated primary LET_f_, with the difference that the calculated μ are shifted to lower values when compared to the ‘4 μm’ (as observed in Figure 9).

**Figure 11:**
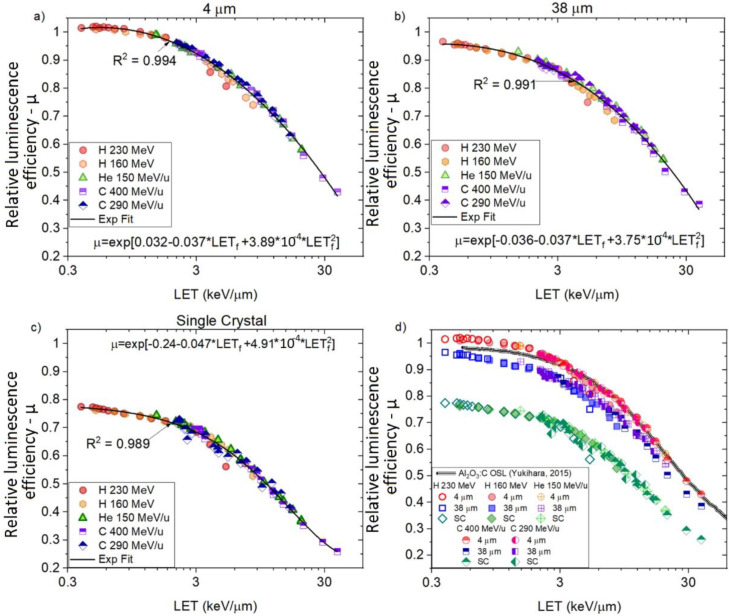
Relative luminescence efficiency (µ) curves and exponential fitting (ExpFit) for (**a**) ‘4 μm’ (R^2^ = 0.994), (**b**) ‘38 μm’ (R^2^ = 0.991) and (**c**) “Single Crystal” (R^2^ = 0.989) acquired from different beam types and energies vs. LET_f_ and (**d**) the combination of all curves plus A_2_O_3_:C OSL (data provided by Dr. Yukihara).

Suppose one uses the fluence weighted contribution of LET_f_ from the primary and each fragment (Equation (11)) instead of using the primary charged particles to plot the relative luminescence efficiencies. In that case, the points in Figure 10 are rearranged in the form presented in Figure 11.

(11) LETf=∑iLETf,i×fluence(%)i i= H1, He4, Li7, Be9, B10, C12, N14, O16

Figure 11a–c show the relative luminescence efficiencies (μ) and fluence weighted LET_f_ (primary + fragments) for ‘4 μm’, ‘38 μm’ and ´Single Crystal´, respectively. In all the plots, the curves of both proton beams (160 MeV and 230 MeV) superimpose (i.e., similar μ for similar averaged LET_f_), smoothly connecting to the curves generated by the helium-charged particles (150 MeV/u) and to the two carbon-charged particles (290 MeV/u and 400 MeV/u). The combination of all curves reveals a trend consistent with previous results for OSL Al_2_O_3_:C [54] (shown in Figure 11d).

A fitting exponential curve can describe the combination of the calculated μ vs. LET_f_ (primary + fragments), as shown in Figure 11a–c by the full black lines. The calculated coefficients of the determination indicate a good correlation, with R^2^ = 0.994 (‘4 μm’), R^2^ = 0.991 (‘38 μm’) and R^2^ = 0.989 (‘Single Crystal’). Based on the results, we observed that (i) each probe presents a unique curve μ and (ii) that μ is independent of beam quality (i.e., only depends on the averaged LET_f_).

### 3.5. Determination of the Birks Factors and Quenching Corrected Curves

**Figure 12:**
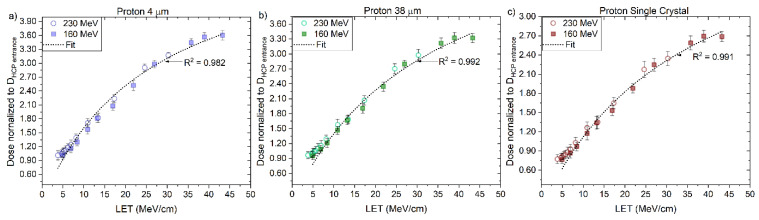
The central axis peak-to-plateau ratios (normalised RL, Figure 5a,b) for the (**a**) ‘4 μm’, (**b**) ‘38 µm’ and (**c**) ´Single Crystal´ fibre probes are shown as a function of averaged electronic stopping power (LET_f_) for mono-energetic proton beams with nominal energies 160 and 230 MeV. The RL_o_ and kB parameters are derived from a nonlinear fit (full line, Equation (3)).

The values of RL_0_ and kB (Table 6) were determined for the ´4 μm´, ´38 μm´ and ‘Single Crystal’ fibre probes irradiated with protons, individually, according to the fitting curves (Equation (3)) presented in Figure 12, as described in Section 2.3. These values are valid for the LET_f_ range from 3 to 45 MeV/cm and used further to correct for quenching using the correction factor derived for proton-charged particles (Equation (8)).

**Figure 13:**
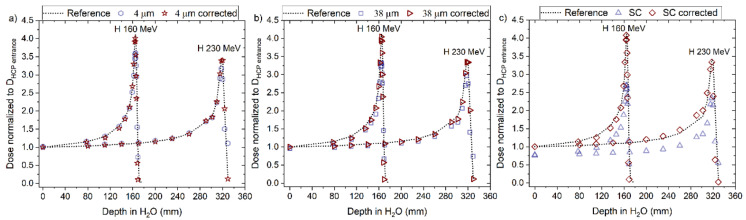
The RL signal converted to dose and normalised to the entrance dose of the reference (D_HCP_ entrance) from the (**a**) 4 µm, (**b**) 38 µm and (**c**) Single Crystal (SC) RL fibre probe before and after the correction factor was applied for 160 and 230 MeV protons beams. The normalised dose from the reference is shown for comparison (dotted line).

**Figure 14:**
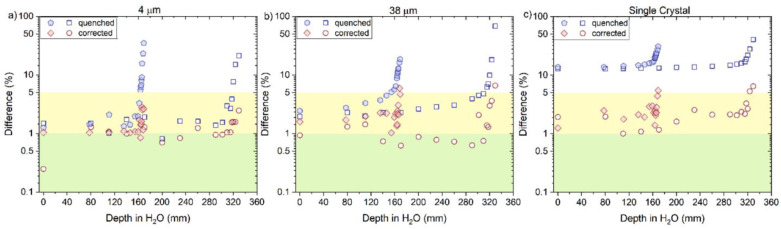
The plots are the difference between the uncorrected (quenched) and corrected RL signal with respect to the reference (ion chamber) from the (**a**) 4 µm, (**b**) 38 µm and (**c**) Single Crystal RL fibre probe before and after the correction factor was applied for 160 and 230 MeV protons beams. The green area in the curve represents the differences < 1%, and the yellow area represents values between 1 and 5%.

The corrected RL measurements for proton beams, using Equation (8), agreed closely with the reference measurements as shown in Figure 13 and Figure 14a–c, for ‘4 μm’, ‘38 μm’ and ´Single Crystal, respectively. A better overall agreement is again observed for ‘4 μm’ compared to the other two probes. The calculated and corrected Bragg peak heights agreed within 3% (‘4 μm’), 4% (‘38 μm’) and 5% (‘Single Crystal’) for both proton beams.

As described in Section 3.3, a good approximation for the correction factor function to be applied to the helium-charged particles is based on Equation (9), where the contribution from the primary beam (^4^He) and the fragment ^1^H account for most of the beam’s fluence.

The Birks factor (kB) is characteristic of the material and can have different values for the same material in different measurements and data treatment conditions. In our study, the kB values were obtained by fitting data for particles of one kind and in some specific energy/LET range. We assumed, as such, that the $kB_{H}$ and $RL_{o_{H}}$ from Equation (9) are the same derived from the fitting in Figure 12 and described in Table 6 for each fibre probe type.

**Figure 15:**
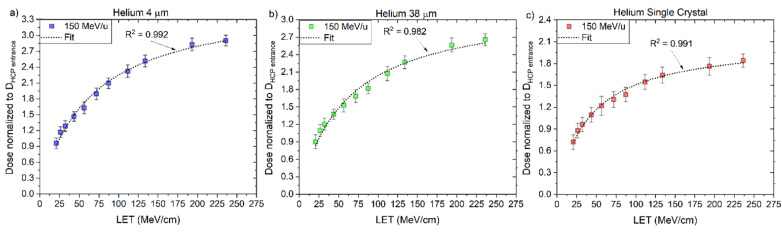
The central axis peak-to-plateau ratios (normalised RL, Figure 5c) for the (**a**) 4 µm, (**b**) 38 µm and (**c**) ´Single Crystal´ fibre probes are shown as a function of averaged electronic stopping power (LET_f_) for mono-energetic proton beams with nominal energies 160 and 230 MeV. A nonlinear fit (full line, Equation (3)) determines the RL_o_ and kB.

The values of RL_0_ and kB (Table 7) were determined for the ‘4 μm’, ‘38 μm’ and ‘Single Crystal’ fibre probes irradiated with 150 MeV/u helium-charged particles, according to the fitting curves (Equation (3)) presented in Figure 15, as described in Section 2.3. These values are valid for the primary LET_f_ range from 20 to 240 MeV/cm from ^4^He. The parameters from Table 6 and Table 7 are combined to correct for quenching using the correction factor derived for helium-charged particles (Equation (9)).

**Figure 16:**
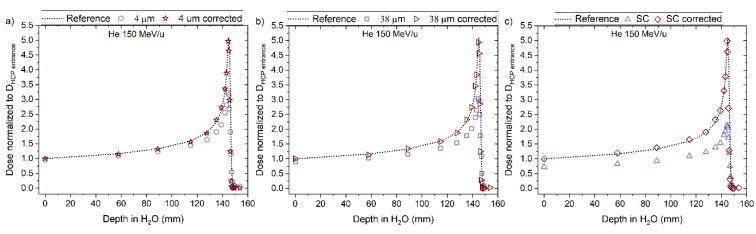
The RL signal converted to dose and normalised to the entrance dose of the reference (D_HCP_ entrance) from the (**a**) 4 µm, (**b**) 38 µm and (**c**) Single Crystal (SC) RL fibre probe before and after the correction factor was applied for 150 MeV/u helium beams. The normalised dose from the reference is shown for comparison (dotted line).

**Figure 17:**
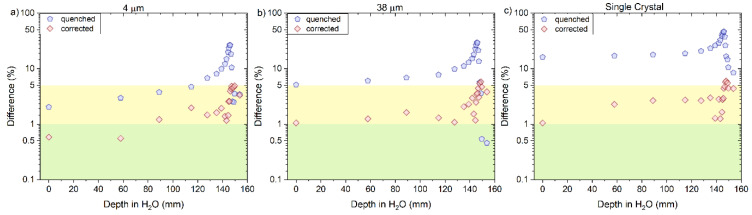
The plots are the difference between the uncorrected (quenched) and corrected RL signal concerning the reference (ion chamber) from the (**a**) 4 µm, (**b**) 38 µm and (**c**) Single Crystal RL fibre probe before and after the correction factor was applied for 150 MeV/u helium beams. The green area in the curve represents the differences < 1%, and the yellow area represents values between 1 and 5%.

The corrected RL measurements for the 150 helium beam, using Equation (9), agreed closely with the reference measurements as shown in Figure 16 and Figure 17a–c, for ‘4 μm’, ‘38 μm’ and ´Single Crystal, respectively. The corrected curves for quenching present a clear improvement in the dose–response, especially for points close to the Bragg peak. For example, the difference between the ‘4 µm’ fibre probe and reference improved from ~30% to ~5% at the Bragg peak (144.91 mm). The same type of improvement is also observed for the other two probes.

Moving forward to correct the quenched curves measured in the carbon-charged particles, Equation (10) is the sum of the contribution from the primary beam (^12^C) and the fragments ^1^H and ^4^He. We assume that the Birks factors (kB) from the fragments are already defined in Table 6 for $kB_{H}$ and $RL_{o_{H}}$, and Table 7 for $kB_{He}$ and $RL_{o_{He}}$ for each fibre probe type.

**Figure 18:**
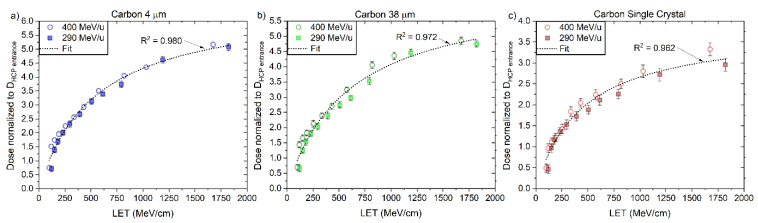
The central axis peak-to-plateau ratios (normalised RL, Figure 5d,e) for the (**a**) 4 μm, (**b**) 38 µm and (**c**) ‘Single Crystal’ fibre probes are shown as a function of averaged electronic stopping power (LET_f_) for mono-energetic carbon beams with nominal energies 290 and 400 MeV/u. The RLo and kB parameters are derived from a nonlinear fit (full line, Equation (3)).

The values of RL_0_ and kB were determined for the ‘4 μm’, ‘38 μm’ and ‘Single Crystal’ fibre probes irradiated with 290 and 400 MeV/u carbon-charged particles, according to the fitting curves (Equation (3)) presented in Figure 18 and Table 8, as described in Section 2.3.

**Figure 19:**
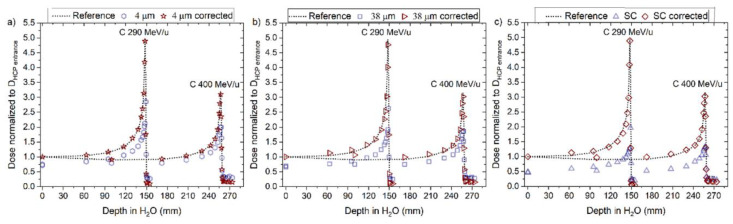
The RL signal converted to dose and normalised to the entrance dose of the reference (D_HCP_ entrance) from the (**a**) 4 µm, (**b**) 38 µm and (**c**) Single Crystal (SC) RL fibre probe before and after the correction factor was applied for 290 and 400 MeV/u carbons beams. The normalised dose from the reference is shown for comparison (dotted line).

**Figure 20:**
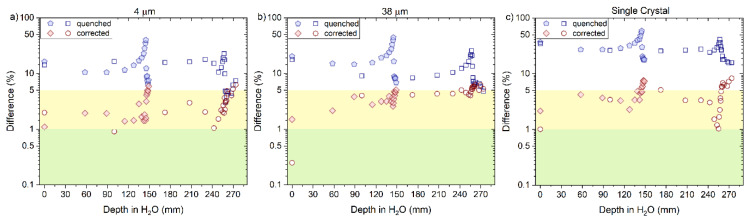
The plots are the difference between the uncorrected (quenched) and corrected RL signal with respect to the reference (ion chamber) from the (**a**) 4 µm, (**b**) 38 µm and (**c**) Single Crystal RL fibre probe before and after the correction factor was applied for 290 and 400 MeV/u carbon beams. The green area in the curve represents the differences < 1%, and the yellow area represents values between 1 and 5%.

The corrected RL measurements for both carbon beams, using Equation (10), resulted in a significant improvement in the difference values concerning the reference dose measurements, as shown in Figure 19 and Figure 20a–c, for ‘4 μm’, ‘38 μm’ and ‘Single Crystal’, respectively. The corrected curves for quenching presented differences with respect to the reference between 5 and 8% for doses around the Bragg peak and in the falloff regions.

## 4. Discussion

In this paper, we studied a method to correct dose quenching in Al_2_O_3_:C RL detectors. The Birks formulation was adapted to account for the contribution of fragments generated along the Bragg curves. Our method has been previously applied to correct for quenching in Al_2_O_3_:C,Mg two-dimensional films irradiated with three different proton and one therapeutic carbon beam. Here, we used our method in three different optical fibre probes and five charged therapeutic beams (protons, helium, and carbon).

For the dose–response test (Figure 3), measured at entrance depth, all the probes presented a linear response for doses up to 2 Gy and at higher doses, supralinearity, with higher deviations observed for the ‘Single Crystal’ probe followed by the ’38 μm’. The results from the ‘4 μm’ fibre probes agreed most with linear dose–response (Figure 4).

The sensitivity of Al_2_O_3_:C detectors changes with irradiation due to the filling of deep electron and hole traps, competing with dosimetric traps during irradiation and readout [55], a phenomenon generally linked to supralinearity. This effect was previously observed in other studies [38,41,56]. Figure 3 suggests a dependency on crystal size and the deposition of energy, similar to those observed for irradiations with other beam types, such as 6 MV photons [38] and heavily charged particles [30,41]. Although identifying the exact mechanisms explaining the differences between crystal sizes needs further analysis, we believe this effect comes from the competition between the immediate recombination of charge carriers and charge trapping. Pre-irradiated fibres stabilised the RL signal from Al_2_O_3_:C [39] and Al_2_O_3_:C, Mg [57]. However, the pre-dosing likely only fills up the charge from the dosimetry traps [58] and does not fill all the deep traps.

If one considers each optical fibre probe as a large cavity, such as the electrons stopping entirely in the RL material, we would not expect differences in quenching observed in probes made with different grain sizes. However, we have observed that the smaller the grain size, the larger the number of ionisations happening in the water-equivalent binder surrounding the grain. In large powder grains and crystals (such as ‘38 μm’ and ‘Single Crystal’), there is a higher absorption of the electrons inside the Al_2_O_3_:C, resulting in a higher ionisation density that causes quenching. This effect with detector size has also been discussed by previous authors using “cavity theory” in OSL/TL passive detectors [59] and scintillators [60] in X-rays.

For measurements along the Bragg curves, we observed quenching for all fibre probes, with a closer agreement to the reference for the ‘4 μm’ fibre, followed by ‘38 μm’ and the ‘Single Crystal’. The same trend was observed for all beams and energies. There is also a better agreement for lower LET beams (i.e., 230 MeV protons) than for the higher LET beams (290 MeV/u). The link between quenching and crystal size was observed previously [30,41], where probes with ‘38 μm’ were compared with ‘Single Crystal’. We further studied crystal size dependence with LET by adding an extra (smaller) crystal size (‘4 μm’) and four extra beams. Although the difference between the rescaled Bragg curves from ‘4 μm’ and ‘38 μm’ was smaller than the difference between ‘38 μm’ and ‘Single Crystal’, we did not find a linear correlation with crystal size.

Quenching was previously observed for Al_2_O_3_:C when used as both passive (OSL) and active (RL) detectors. Andersen et al. studied the Al_2_O_3_:C RL vs. absorbed dose–rate during 175 MeV proton radiotherapy [61]. They observed that in the low 0–0.3 Gy range, the RL signal closely resembles that observed for a clinical 6 MV X-ray beam without any LET-dependent correction factors. In contrast, the relative luminescence efficiency decreased to about 60% for higher doses. Klein et al. tested a thin layer of Al_2_O_3_:C to resolve the steep gradients of the ion depth-dose curves in 142.66 MeV proton and 270.55 MeV/u carbon ion beams and observed a relative luminescence efficiency dropping for higher LET values [62]. Measurements with helium, carbon, neon and iron ions demonstrated that the Al_2_O_3_:C OSL signal is also strongly LET-dependent [34,41].

The energy deposition along the Bragg curves can explain the quenching dependence with LET. Near the Bragg Peak and in the falloff region, primary proton, helium, and carbon-charged particles experience a rapid increase in their LET values, nearing the end of their ranges. Fragmentation (most prominent for carbon beams) generates secondary particles with very high LET (such as alpha particles and heavy ion recoils) [63] that will create regions of highly high local dose in the close vicinity of the ion track, saturating RL centres and causing luminescence quenching [64]. As scintillators are used in several applications where heavy particles are present, from medical applications to dark matter studies, many approaches for the calculation of quenching factors have been proposed [43,65,66,67]. However, there is no standard theory to predict and describe measured quenched response curves. According to the Birks model, two ions with the same LET but a different atomic number (Z) will result in the same ionisation quenching and, consequently, the same kB. Many experiments, however, contradict such a statement [67,68,69], showing that the Birks factor (kB) is characteristic of the material and can have different values for the same material in various measurements and data treatment conditions.

Here, the kB values were obtained by fitting data for particles of one kind and in some specific energy/LET range. The fitting curves derived from the measured ‘4 μm’ RL signals and simulated LET_f_ values are R^2^ > 0.982 for protons, R^2^ > 0.992 for helium and R^2^ > 0.991 for carbon-charged particles (Figure 12a, Figure 15a and Figure 18a), from the measured ‘38 μm’ RL signals and simulated LET_f_ values, are R^2^ > 0.992 for protons, R^2^ > 0.982 for helium and R^2^ > 0.991 for carbon-charged particles (Figure 12b, Figure 15b and Figure 18b) and from the measured ‘Single Crystal’ RL signals and simulated LET_f_ values are R^2^ > 0.980 for protons, R^2^ > 0.972 for helium and R^2^ > 0.962 for carbon-charged particles (Figure 12c, Figure 15c and Figure 18c).

Our method shows promising results when applied in the plateau and peak region of the Bragg curves for the five beam types studied. The method is less accurate for the points measured at falloff (see Section 3.5). We believe this is due to the low doses measured and the worse statistics from the Monte Carlo simulations in this region. To improve these errors, we recommend a dedicated measurement campaign, using much higher doses to improve signal-to-noise ratios and new Monte Carlo simulations with a more significant number of events.

## 5. Conclusions

We found that the response of Al_2_O_3_:C RL detectors is LET_f_-dependent, a general phenomenon observed in solid-state dosimeters. Because of the decrease in relative luminescence efficiencies with LET, doses in heavily charged particle beams cannot be calculated directly from real time RL measurements unless in regions and energies where the relative luminescence efficiencies are flat.

The RL response from three types of Al_2_O_3_:C optical fibre probes to radiotherapy proton, helium, and carbon-charged particles have been investigated and compared with ionisation chamber measurements in the same conditions. The observed LET-related quenching under response along the Bragg curve was corrected using a new method based on RL measurements and Monte Carlo simulated fluence averaged-LET values. This method demonstrated the linear dose response of all the optical fibre probes.

## Figures and Tables

**Table 1:** Details of each measurement campaign at HIMAC with types and energies of beams, dose rates, and types and quantities of fibre probes.

Beam Type and Energy [MeV/u]	Dose Rate [Gy/min]	Fibber Probes (Quantity)	Dose to Water at Entrance (d = 0 mm H_2_O)
**H 160**	0.08 (a) 0.25 (b)	Single Crystal (1) 38 μm (1) 4 μm (1)	**Dose–response**: from 0.04 to 0.10 Gy (a) from 0.1 to 5.0 Gy (b) **Bragg curve**: 0.05 Gy (a) 0.25 Gy (b)
**H 230**	0.36 (a) 0.49 (b) 0.65 (c)	Single Crystal (1) 38 μm (2) 4 μm (2)	**Dose–response**: from 0.04 to 0.50 Gy (a), from 0.06 to 2.00 Gy (b) from 0.5 to 2.0 Gy (c) **Bragg curve**: 0.5 and 1.0 Gy (a) 0.05 and 0.50 Gy (b) 0.5 and 1.0 Gy (c)
**He 150**	4.50	Single Crystal (1) 38 μm (2) 4 μm (2)	**Dose–response**: 0.3 to 10.0 Gy **Bragg curve**: 0.5, 1.0, and 2.0 Gy
**C 290**	6.90 (a) 7.34 (b) 3.04 (c)	Single Crystal (1) 38 μm (2) 4 μm (2)	**Dose–response**: 0.5 to 10.0 Gy (a) and (c) 5 to 60 Gy (b), **Bragg curve**: 0.5 and 1.0 Gy (a) and (c) 1, 2 and 5 Gy (b)
**C 400**	6.90	Single Crystal (1) 38 μm (1) 4 μm (1)	**Dose–response**: 0.1 to 60.0 Gy **Bragg curve**: 0.5, 2.0 Gy

**Table 2:** Relative luminescence efficiency of fibre probes (‘Single Crystal’, “38 μm” and “4 μm”) at entrance depth in H_2_O for H 230, H 160, He 150, C 400, and C 290.

Energy (MeV/u)	LET (keV/μm)	*Relative Luminescence Efficiency (μ)* *at Entrance Depth in H_2_O*		
		4 μm	38 μm	Single Crystal
**H 230**	0.41	1.02 ± 0.03	0.97 ± 0.03	0.77 ± 0.06
**H 160**	0.54	1.01 ± 0.02	0.96 ± 0.03	0.76 ± 0.07
**He 150**	2.25	0.95 ± 0.02	0.90 ± 0.02	0.71 ± 0.07
**C 400**	11.22	0.75 ± 0.04	0.71 ± 0.03	0.56 ± 0.08
**C 290**	13.50	0.73 ± 0.03	0.69 ± 0.02	0.55 ± 0.08

**Table 3:** Fluence and LET_f_ were simulated for the 160 MeV and 230 MeV primary beam (^1^H) and two fragments (^4^He and ^7^Li) for four depths in water: entrance, end of the f plateau, Bragg peak, and falloff.

Depth in H_2_O (mm)	Fluence (%)	LET_f_ (keV μm^−1^)	Fluence (%)	LET_f_ (keV μm^−1^)	Fluence (%)	LET_f_ (keV μm^−1^)
	^1^H		^4^He		^7^Li	
	230 MeV					
**00.10**	99.999	0.381	3.6 × 10^−3^	83.62	4.5 × 10^−5^	502.442
**170.42**	99.994	0.567	4.2 × 10^−3^	82.92	4.1 × 10^−5^	490.233
**317.74**	99.994	3.01	4.1 × 10^−3^	122.07	3.3 × 10^−6^	519.452
**329.4**	99.981	6.987	12.8 × 10^−3^	115.18	2.3 × 10^−4^	575.503
	160 MeV					
**00.10**	99.995	0.496	3.4 × 10^−3^	84.60	3.9 × 10^−5^	536.542
**110.8**	99.995	0.842	3.8 × 10^−3^	87.27	4.6 × 10^−5^	528.111
**164.81**	99.996	3.820	2.7 × 10^−3^	127.22	2.7 × 10^−5^	555.512
**168.8**	99.997	7.93	1.7 × 10^−3^	104.29	7.7 × 10^−5^	583.401

**Table 4:** Fluence and LET_f_ simulated for the 150 MeV/u primary beam (^4^He) and two fragments (^1^H and ^7^Li) for four depths in water: entrance, end of plateau, Bragg peak, and falloff.

Depth in H_2_O (mm)	Fluence (%)	LET_f_ (keV μm^−1^)	Fluence (%)	LET_f_ (keV μm^−1^)	Fluence (%)	LET_f_ (keV μm^−1^)
	^4^He		^1^H		^7^Li	
	150 MeV/u					
**00.10**	99.636	2.077	0.360	3.94	4.00 × 10^−4^	423.292
**88.91**	74.705	3.221	25.255	1.241	3.30 × 10^−4^	27.360
**144.91**	70.525	22.659	29.471	1.562	6.29 × 10^−5^	205.123
**148.1**	1.227	56.400	98.768	1.464	1.49 × 10^−5^	330.995

**Table 5:** Fluence and LETf simulated for the 400 MeV/u and 290 MeV/u primary beam (^12^C) and two fragments (^1^H and ^4^He) for four depths in water: entrance, end of plateau, Bragg peak and falloff.

Depth in H_2_O (mm)	Fluence (%)	LET_f_ (keV μm^−1^)	Fluence (%)	LET_f_ (keV μm^−1^)	Fluence (%)	LET_f_ (keV μm^−1^)
	^12^C		^1^H		^4^He	
	400 MeV/u					
**00.10**	98.196	9.883	1.646	2.510	0.117	16.517
**172.24**	18.948	14.972	56.712	0.636	18.215	1.494
**256.9**	8.031	167.429	61.438	0.763	23.790	2.455
**259.21**	0.054	291.223	66.900	0.740	25.929	2.411
	290 MeV/u					
**00.10**	98.600	11.985	1.237	2.790	0.110	15.473
**95.03**	32.500	18.089	45.839	0.836	15.946	1.875
**147.92**	18.900	182.151	50.947	1.036	23.109	3.366
**150.15**	0.0001	170.56	63.010	0.981	28.714	3.278

**Table 6:** Comparison of the determined kB and RL_o_ parameters, as in Equation (3), for ‘4 µm’, ‘38 µm’ and ‘Single Crystal’ for proton-charged particles.

Beam Type/Energy (MeV/u)	LET_f_ (MeV cm^−1^)	kB (μg MeV^−1^ cm^−2^)			$RL_{0}$		
		4 μm	38 μm	Single Crystal	4 μm	38 μm	Single Crystal
H 230 H 160	3–45	(300 ± 20)	(300 ± 30)	(250 ± 30)	1.13	1.09	0.856

**Table 7:** Comparison of the determined kB and RL_o_ parameters, as in Equation (3), for 4 µm, 38 µm and Single Crystal for helium-charged particles.

Beam Type/Energy (MeV/u)	LET_f_ (MeV cm^−1^)	kB (μg MeV^−1^ cm^−2^)			$RL_{0}$		
		4 μm	38 μm	Single Crystal	4 μm	38 μm	Single Crystal
He 150	20–240	(120 ± 30)	(110 ± 35)	(100 ± 42)	1.3	1.2	0.78

**Table 8:** Comparison of the determined kB and RL_o_ parameters, as in Equation (3), for 4 µm, 38 µm and Single Crystal for carbon-charged particles.

Beam Type/Energy (MeV/u)	LET (MeV cm^−1^)	kB (μg MeV^−1^ cm^−2^)			$RL_{0}$		
		4 μm	38 μm	Single Crystal	4 μm	38 μm	Single Crystal
C 400 C 290	90–1700	(1.8 ± 0.2)	(1.7 ± 0.3)	(2.5 ± 0.4)	0.2	0.19	0.06

## Data Availability

Data will be made available on request.
